# IL-4/IL-4 Ab complex enhances the accumulation of both antigen-specific and bystander CD8 T cells in mouse lungs infected with influenza A virus

**DOI:** 10.1186/s42826-023-00183-2

**Published:** 2023-12-01

**Authors:** Hi Jung Park, Eun Ah Choi, Sung Min Choi, Young-Ki Choi, Jae Il Lee, Kyeong Cheon Jung

**Affiliations:** 1 Graduate Course of Translational Medicine, Seoul National University College of Medicine, Seoul, 03080 Republic of Korea; 2 Transplantation Research Institute, Seoul National University College of Medicine, Seoul, 03080 Republic of Korea; 3 Department of Medicine, Seoul National University College of Medicine, Seoul, 03080 Republic of Korea; 4 Department of Microbiology, College of Medicine and Medical Research Institute, Chungbuk National University, Cheongju, Chungcheongbuk-do 28644 South Korea; 5 Department of Pathology, Seoul National University College of Medicine, Seoul, 03080 Republic of Korea; 6 Integrated Major in Innovative Medical Science, Seoul National University Graduate School, Seoul, 03080 Republic of Korea

**Keywords:** Interlukin-4, Virtual memory, CD8 T cells, CXCR3, Influenza

## Abstract

**Background:**

Unlike conventional T cells, innate and virtual-memory CD8 T cells in naïve mice acquire their memory phenotypes and functions in the absence of antigenic encounters in a cytokine-dependent manner. The relevant cytokines include interleukin-4 (IL-4), type I interferon, and interleukin-15 (IL-15). Moreover, exogenous IL-4 can also induce de novo generation and/or expansion of the virtual-memory CD8 T cell population. In this study, we investigated whether exogenous IL-4 could enhance the immune response to a viral infection.

**Results:**

In vivo administration of IL-4 and an anti-IL-4 antibody complex (IL-4C) increased CXCR3 expression in both memory and naïve phenotype CD8 T cells in the absence of antigenic stimulation, and protected mice from lethal influenza infection. Flow cytometric analysis of lung-infiltrating immune cells on day 5 after virus infection revealed higher numbers of antigen-specific and bystander CD8 T cells in IL-4C-treated mice than in control mice. In particular, the bystander CD8 T cells were a naïve or evident memory phenotypes. Crucially, an anti-CXCR3 blocking antibody abrogated this IL-4C effect, reflecting that the increased accumulation of CD8 T cells in the lungs after IL-4C treatment is dependent on CXCR3.

**Conclusions:**

These data demonstrate that exogenous IL-4C plays a protective role by enhancing CXCR3-dependent migration of CD8 T cells into influenza-infected lungs.

**Supplementary Information:**

The online version contains supplementary material available at 10.1186/s42826-023-00183-2.

## Background

Conventional memory T cells of both humans and mice are generated from naïve T cells when the cells encounter antigens in peripheral lymphoid organs, followed by cell activation and differentiation. However, some T cells of the thymus and peripheral tissues attain the memory phenotype, although they have never encountered antigens. The prototypes of such antigen-inexperienced memory-like T cells (termed innate T cells) are selected by MHC class Ib molecules during thymocyte development and include natural killer T (NKT) cells, CD8αα^+^ intra-epithelial T cells, mucosal-associated invariant T (MAIT) cells, and H2-M3-specific CD8 T cells [1–3]. In addition, the classic MHC class I- or II-restricted selection pathways induce other innate T cells, including T-T CD4 T cells, Eomes^+^ innate CD8 T cells, and natural Th1 cells in the thymus [4–8]. Unlike MHC class Ib-restricted innate T cells, such innate T cells exhibit a diverse TCR repertoire. The development of Eomes^+^ innate CD8 T cells requires interleukin (IL)-4 or type I interferon [3, 6, 7, 9].

Antigen-inexperienced memory-like CD8 T cells are present in the periphery of unimmunized mice including germ-free mice [10]. Such cells are phenotypically similar to Eomes^+^ innate CD8 T cells [11]. These virtual-memory T cells develop under lymphopenic conditions or are induced by certain cytokines, including IL-4, IL-15, and type I interferon [11–14]. IL-4 administration to mice triggers the development and expansion of the innate/virtual-memory CD8 T cell population [15]. Previously, we found that IL-4-induced innate CD8 T cells facilitated rapid viral clearance because they evidenced fast early proliferation and effector T cell generation in leukocyte choriomeningitis virus (LCMV) clone-13-infected mice [16].

CXCR3 is a chemokine receptor that has a significant impact on T cell trafficking and function [17]. This chemokine receptor has been reported to be highly expressed in IL-4-induced CD8 innate T cells, along with CD44, CD122, and Eomes [6]. Furthermore, peripheral CD8 T cells reduced in IL-4Rα KO mice exhibited a CD44^hi^CXCR3^+^ phenotype [18]. Conversely, the administration of exogenous IL-4/IL-4 Ab complex to mice resulted in the development and expansion of CD44^hi^CXCR3^+^ CD8 T cell populations in both thymus and peripheral lymphoid organs [15, 19, 20]. We thus explored whether exogenous IL-4/IL-4 Ab complex could enhance the antiviral CD8 T cell response in influenza-infected mice.

## Results

### IL-4 and the anti-IL-4 complex protected mice from lethal influenza A infection

**Fig. 1:**
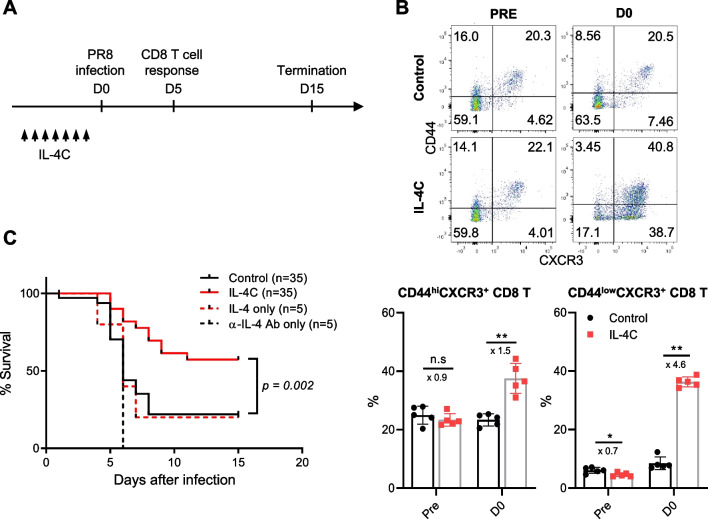
IL-4 and anti-IL-4 antibody complex (IL-4C) treatment protects mice from lethal influenza A virus infection. IL-4 (1.5 µg) and anti-IL-4 antibody (7.5 µg) were mixed and injected intraperitoneally into mice daily for 7 days. On day 8, PBS-treated control and IL-4C-treated mice were intranasally infected with the PR8 strain of influenza A virus. **A** The experimental protocol. **B** Peripheral blood cells were collected from control and IL-4C-treated mice before (Pre) and after (D0) PBS or IL-4C administration, and the expression levels of CD44 and CXCR3 in CD8 T cells were analyzed by flow cytometry. The numbers in the dot plots are the percentages of cells in each quadrant. The data are the means ± SDs (n = 5/group); the x-numbers indicate fold changes. **C** The survival of influenza-infected mice was monitored daily for 15 days for the PBS control (n = 35), IL-4C (n = 35), IL-4-only (n = 5), and anti-IL-4 Ab-only treated groups (n = 5). Statistical analysis was measured using an unpaired t-test and Mantel–Cox test. n.s, not significant. *p < 0.05; ***p < 0.001

To evaluate the effect of IL-4 on both antigen-specific and bystander CD8 T cells, IL-4 complexed with anti-IL-4 Ab (IL-4C) was injected daily for 7 days. Subsequently, we infected the mice with influenza. The phenotypic changes in peripheral blood CD8 T cells were examined via flow cytometry and the mice were intranasally infected with a lethal dose (6 × 10^3^ pfu/mouse) of PR8 influenza virus (Fig. 1A). As reported previously (15, 20), we found that intraperitoneal IL-4C injection increased the blood numbers of CD44^hi^CXCR3^+^ and CD44^low^CXCR3^+^ CD8 T cells (Fig. 1B). Moreover, the survival rate of PR8-infected mice was significantly higher in the IL-4C-treated group than in the phosphate buffered saline (PBS)- or anti-IL-4 Ab-treated groups. IL-4 injection alone did not protect mice from lethal influenza (Fig. 1C). As injected free IL-4 is only transiently active, unlike IL-4C [21], the data suggest that the improved survival of IL-4C-treated mice reflected the in vivo bioactivity of IL-4.

### IL-4 and the anti-IL-4 complex enhanced CD8 T cell accumulation in influenza-infected lungs

**Fig. 2:**
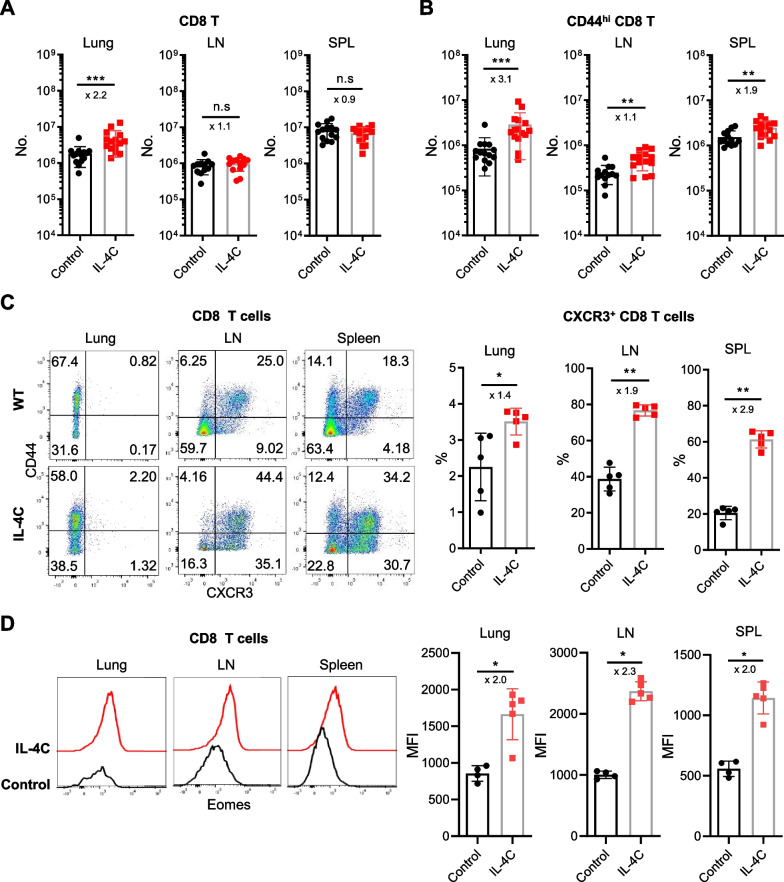
IL-4C enhances CD8 T cell accumulation and CXCR3 and Eomes expression in CD8 T cells. On day 5 after influenza infection, lymphocytes were harvested from the lungs, peribronchial lymph nodes (LNs), and spleens, and CD8 T cells were analyzed via flow cytometry. Total (**A**) and CD44^hi^ memory-phenotype (**B**) CD8 T cell numbers were counted. The results are the means ± SDs (n = 15/group); the x-numbers indicate fold changes. The percentage of CXCR3^+^ CD8 cells (**C**) and the mean fluorescence intensity (MFI) of Eomes expression in CD8 T cells (**D**). Representative plots are shown on the left; the numbers in the dot plots are the cell percentages in each quadrant. The summarized data are the means ± SDs (n = 5/group); the x-numbers are the fold changes. Statistical analysis was measured using an unpaired t-test. n.s, not significant. *p < 0.05; **p < 0.01; ***p < 0.001

The lungs, lymph nodes, and spleens of infected mice were harvested 5 days after PR8 infection and the CD8-expressing T cells were analyzed via flow cytometry. There were twice as many CD8 T cells in the lungs of IL-4C-treated mice as in the controls (*p* < 0.01), but lymph node and spleen numbers did not significantly differ (Fig. 2A). IL-4C increased the numbers of CD44^hi^ CD8 T cells in all organs (Fig. 2B); as expected, the proportion of CXCR3^+^ CD8 T cells was higher in IL-4C-treated mice than in controls (Fig. 2C). On a per cell basis, Eomes expression in CD8 T cells, which is associated with effector/memory functions, was also upregulated in IL-4C-treated mice (Fig. 2D).

**Fig. 3:**
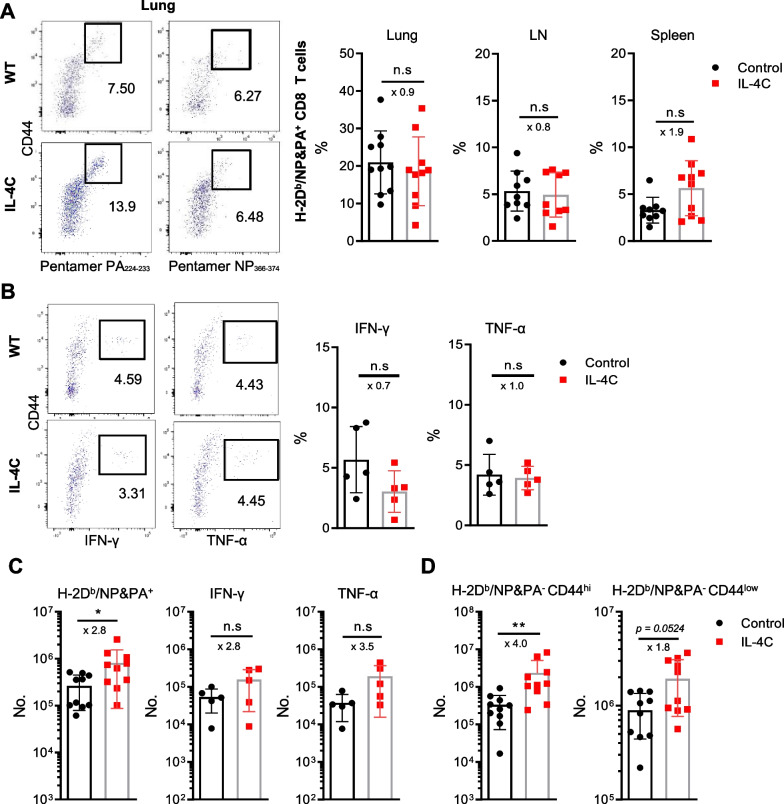
Influenza-specific and bystander CD8 T cell numbers are higher in IL-4C-treated mice than in controls. Cells were isolated from lungs, peribronchial lymph nodes (LNs), and spleens on day 5 after influenza infection. **A** CD8 T cells from each organ were stained with H-2D^b^/NP_366–374_ and H-2D^b^/PA_224–233_ pentamers (H-2D^b^/NP&PA) and analyzed via flow cytometry. Representative dot plots and the summarized proportions of virus-specific CD8 T cells are shown. The numbers in the dot plots are pentamer^+^ cell percentages. **B** Cells from lungs were restimulated ex vivo with NP_366–374_ peptides in the presence of monensin for 5 h and then stained with anti-IFN-γ or TNF-α antibodies. Representative dot plots and the summarized proportions of cytokine-producing CD8 T cells are shown. The numbers in the dot plots are cytokine-producing cell percentages. **C** Flow cytometry yielded the antigen-specific (pentamer-, IFN-γ-, or TNF-α-positive) and **D** bystander (pentamer-negative CD44^hi^ or CD44^low^) CD8 T cell numbers. The data are the means ± SDs (n = 5 or 10/group); the x-values are fold changes. Statistical analysis was performed using an unpaired t-test. n.s, not significant. *p < 0.05; **p < 0.01

Next, we investigated whether IL-4C expanded the populations of antigen-specific effector/memory CD8 T cells. CD8 T cells were stained with H2-D^b^ pentamers containing NP366–374 and PA224–233 peptides. As shown in Fig. 3A, NP366–374 and PA224–233 peptide-specific CD8 T cell proportions in the lungs and lymph nodes did not differ between IL-4C-treated mice and controls (refer to the dot plot of LN and spleen in Additional file 1: Fig. S1). When influenza-specific CD8 T cell responses were assessed via ex vivo restimulation with influenza NP366–374 peptides, the proportions of interferon-γ (IFN-γ)- or tumor-necrosis factor-α (TNF-α)-producing lung CD8 T cells were comparable between control and treated mice (Fig. 3B). However, the lungs of IL-4C-treated mice had approximately threefold more influenza virus-specific CD8 T cells (as revealed by pentamer-binding or ex vivo cytokine production), although the differences in cytokine-producing cell numbers did not attain statistical significance (Fig. 3C). Moreover, the numbers of both pentamer-negative CD44^hi^ memory-phenotype and CD44^low^ naïve-phenotype CD8 T cells in the lungs were significantly increased by IL-4C treatment (*p* < 0.05; Fig. 3D). Notably, the differences in cell number between the two groups were greatest for the pentamer-negative, CD44^hi^ memory-phenotype cell population, most of which were bystander memory CD8 T cells. Together, these data indicate that IL-4C enhanced the accumulation of both antigen-specific and bystander CD8 T cells in infected lungs.

### Increased accumulation of CD8 T cells in influenza-infected lungs after IL-4C treatment is dependent on CXCR3

**Fig. 4:**
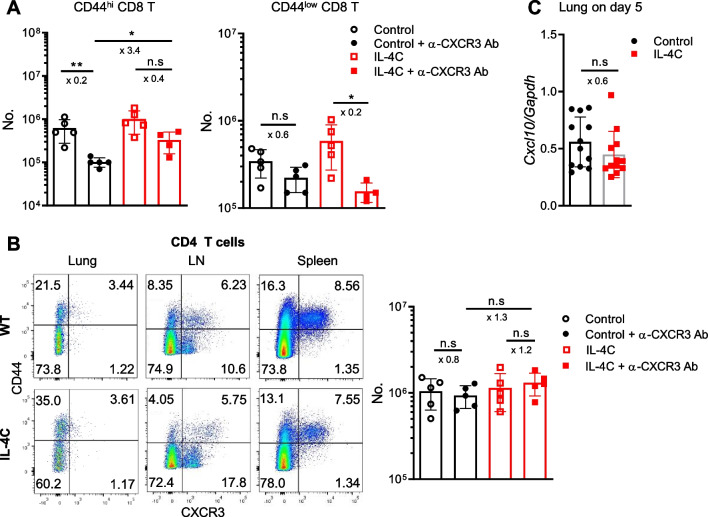
Anti-CXCR3 antibody inhibits IL-4C-induced CD8 T cell accumulation in the lungs. After IL-4C injection daily for 7 days, control and IL-4C-treated mice were intraperitoneally injected with 500 µg amounts of anti-CXCR3 Ab, followed by influenza A infection. Lungs were harvested 5 days after infection. **A** CD44^hi^ and CD44^low^ CD8 T cell numbers were determined via flow cytometry. The data are the means ± SDs (n = 4–5/group). **B** CD44^hi^ and CD44^low^ CD4 T cell numbers were determined. The data are the means ± SDs (n = 5/group). (**C**) *Cxcl10* transcript levels were measured using real-time PCR and normalized to the expression levels of the *Gapdh* gene. The data are means ± SDs (n = 12/group); the x-values are fold changes. n.s, not significant. Statistical analysis was performed using an unpaired t-test. *p < 0.05; **p < 0.01

To explore the mechanism underlying the enhanced accumulation of CD8 T cells in infected lungs after IL-4C treatment, we focused on the CXCR3 protein that recruits antigen-specific CD8 T cells to the lungs during respiratory virus infection [22, 23]. Anti-CXCR3-blocking antibodies were intraperitoneally injected into control and IL-4C-treated mice, followed by intranasal PR8 infection (on the same day). On day 5 post infection, CD8 T cell numbers and phenotypes were analyzed. As expected, the anti-CXCR3 antibody reduced the effect of IL-4C on the accumulation of both CD44^hi^ and CD44^low^ cells (Fig. 4A), reflecting CXCR3 upregulation (in both cell populations) by IL-4C (Fig. 2C). However, CXCR3 expression in CD4 T cells and CD4 T cell infiltration into infected lungs were not affected by IL-4C (Fig. 4B). Furthermore, transcription of the *Cxcl10* gene, which encodes the principal CXCR3 ligand in influenza-infected lungs [24], was not affected by IL-4C (Fig. 4C), suggesting that IL-4-driven CXCR3 upregulation in both CD44^hi^ and CD44^low^ CD8 T cells caused such cells to accumulate in inflamed lungs.

**Fig. 5:**
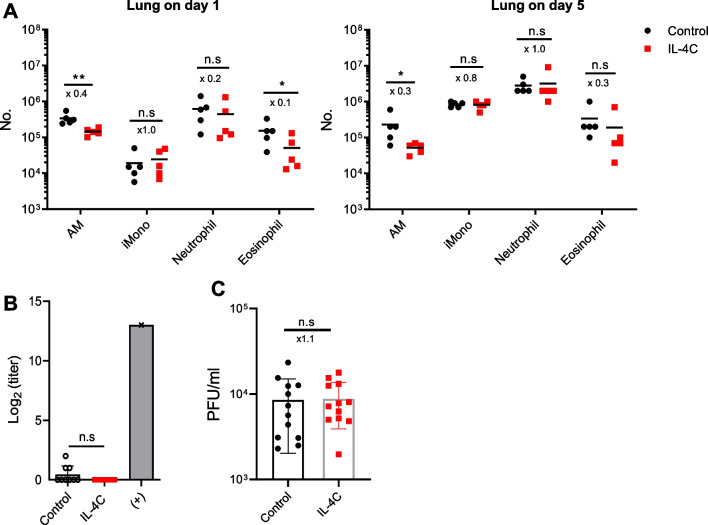
IL-4C has no impact on innate cell infiltration or antibody titer. Cells were isolated from the lungs of IL-4 treated and control mice. **A** The numbers of innate immune cells (alveolar macrophage (AM), inflammatory monocyte (iMono), neutrophil and eosinophil) were counted via flow cytometry on days 1 and 5 after influenza A infection. The data are the means ± SDs (n = 5/group); the x numbers indicate fold changes. **B** Sera of IL-4-treated and control mice were collected 5 days after influenza A infection, and the anti-PR8 antibody titers were assessed via ELISA. Sera obtained 20 days after influenza infection of naïve mice served as the positive (+) control. **C** Virus titers in the lungs of each group were analyzed by plaque assay. A pooled graph is shown (n = 12/group). The x-values represent fold change. The data are the means ± SD (n = 9/group). Statistical analysis was performed using an unpaired t-test and Mann–Whitney test. n.s, not significant. *p < 0.0; **p < 0.01

Next, we assessed whether IL-4C affected the early innate immune response. The numbers of neutrophils, inflammatory monocytes and eosinophils did not differ on day 1 or 5 after PR8 infection between the IL-4C and control groups, although the numbers of alveolar macrophages were lower in the IL-4C-treted group (Fig. 5A). The anti-PR8 HA antibody titers in sera of control and IL-4C-treated mice were negligible on day 5 after influenza infection, suggesting that such antibodies did not contribute to the survival improvement of treated mice at the early time of influenza infection (Fig. 5B). Similarly, no significant differences in virus titer were observed in the lung (Fig. 5C).

## Discussion

Here, we protected mice from lethal influenza infection by administering IL-4 in complex with anti-IL-4 Ab. As expected, IL-4C injection increased the number of influenza-specific CD8 T cells in infected lungs. However, the increase principally featured bystander CD44^hi^ CD8 T cells that could not recognize the influenza NP and PA epitopes. Moreover, the number of CD44^low^ naïve phenotype CD8 T cells was also higher in the lungs of treated mice. We further found that augmented infiltration of both virus-specific and bystander CD8 T cells was dependent on CXCR3, the expression of which was upregulated in both naïve and memory CD8 T cells in response to IL-4C.

CXCR3 is a key chemokine receptor for migration into sites of inflammation, and is rapidly upregulated in CD8 T cells following antigen recognition and cell activation via interaction with dendritic cells [17, 25, 26]. Three CXCR3 ligands are known: CXCL9 (monokine induced by γ-interferon, MIG), CXCL10 (interferon-induced protein of 10 kDa, IP-10), and CXCL11 (interferon-induced T cell α chemoattractant, I-TAC). The expression of these ligands at sites of inflammation is induced by IFN-γ; all elicit migration of CXCR3-expressing cells [27]. In influenza-infected lungs, CXCL10 is the major CD8 T cell recruiter [24]. Previously, we showed that CXCR3^+^ cells in mice constitute approximately 60% of memory CD8 T cells and that IL-4C increased that proportion to approximately 80% in the absence of antigenic stimulation [15]. Moreover, a similar effect was evident in terms of CD44^low^ naïve phenotype CD8 T cells. Only approximately 10% of CD44^low^ CD8 T cells in the spleens of naïve mice expressed surface CXCR3; this increased to 85% in IL-4C-treated mice. Thus, IL-4C enhanced the migration of both antigen-specific and bystander CD8 T cells into inflamed lungs after influenza infection by triggering antigen-independent upregulation of CXCR3 expression in CD8 T cells.

Most IL-4-induced innate CD8 T cells express CXCR3 [7]; such cells enhance immunity against bacterial infection and cancer [28]. CXCR3^+^ naïve CD8 T cells are also functionally superior to CXCR3^−^ naïve cells in terms of cytokine production and effector function [29]. Thus, it may be that IL-4-induced CXCR3 upregulation in both memory and naïve T cells contributes to antitumor immunity. However, this has not been confirmed, although IL-4 has potent antitumor effects in several mouse models [30–32].

In this study, IL-4C treatment had no effect on the expression of CXCR3 on CD4 T cells and their migration into the inflamed lung, in contrast to CD8 T cells. While approximately 20% of splenic CD8 T cells in naïve mice exhibit a CXCR3^+^ virtual memory phenotype [15], only a very small population of CD4 T cells in naïve mice displays a similar phenotype. It remains undocumented whether the CXCR3^+^ memory phenotype CD4 T cells in naïve mice are the counterparts of virtual memory CD8 T cells. Furthermore, CXCR3 expression on CD4 T cells is associated with Th1 differentiation, which is typically IL-12 and IFN-γ rather than IL-4 dependent [17]. Taking these, it appears that IL-4C has a limited effect on CXCR3 expression in CD4 T cells compared to CD8 T cells, although the specific mechanism underlying this difference has not been elucidated.

In this study, we did not observe any significant differences in viral titers and antiviral antibody levels between IL-4-pretreated mice and the control group. These findings suggest that IL-4C treatment did not improve the survival of influenza-infected mice by enhancing the antiviral antibody response. Additionally, the improved survival rate observed in IL-4C-treated mice following influenza infection does not appear to be solely due to enhanced effector function of CD8 T cells. Severe influenza infection is associated with lung epithelial damage due to excessive infiltration of inflammatory cells, particularly neutrophils and monocytes [33]. Previous studies have reported that IL-4 administration alleviates lung damage via macrophage reprogramming in models of acute lung injury [34]. In addition, IL-4 promoted lung epithelial cell regeneration in sepsis-induced acute lung injury by polarizing macrophages to the M2 type. IL-4 has also been shown to attenuate neutrophil migration into inflamed tissue [35, 36]. IL-4Rα signaling suppresses acute lung injury by inhibiting neutrophil survival [37]. Given these findings, it is plausible that exogenous IL-4 treatment may prevent lung injury by inducing macrophage M2 polarization and/or limiting neutrophil infiltration or survival, although no significant differences were found in the number of macrophages and neutrophil in infected lungs between IL-4C-treated and control mice.

## Conclusions

Exogenous IL-4 protected mice against lethal influenza infection. Such treatment markedly increased CXCR3 expression in both memory and naïve CD8 T cells, enhancing the migration of both antigen-specific and bystander CD8 T cells into inflamed lungs after infection. Our results afford new insight into the role played by IL-4-induced virtual- memory CD8 T cells in antiviral immunity.

## Methods

### Mice, infection, and ethics

All mice were maintained under a 12:12 light–dark cycle in specific pathogen free conditions at animal care facilities of the Biomedical Center for Animal Resource Development of Seoul National University and the Seoul National University Hospital Biomedical Research Institute under specific pathogen free conditions. All mice were housed in groups of as many as 5 mice per cage with a temperature set point of 22 ± 1 °C and humidity of 50 ± 10%. C57BL/6 mice were purchased from Koatech (Pyeongtaek, Korea). As described previously [15, 20], 1.5 µg mouse IL-4 (Peprotech, Princeton, NJ, USA) and 7.5 µg antibody against mouse IL-4 (11B11; Bio X Cell, West Lebanon, NH, USA) were mixed in 200 μL PBS and intraperitoneally injected into mice daily for 7 days. Control mice received only PBS, IL-4, or anti-IL-4 Ab. One day after the final injection, mice were anesthetized with isoflurane and infected with influenza A/Puerto Rico/8/34 (H1N1, PR8) virus intranasally; animal health and behavior were checked every day up to 15 days after infection. Mice were euthanized if they showed the following symptoms and signs: abnormal respiration, significant lethargy, or moribund appearance. In some experiments, mice were sacrificed after 5 days of infection, and the immune cell profiles of the lungs, lymph nodes, and spleens were assessed via flow cytometry.

### Flow cytometric analysis

Single-cell suspensions of lungs, lymph nodes, and spleens were prepared. Lungs were placed in 5 mL of RPMI-1640 medium with 1 mg/mL type IV collagenase (Worthington Biochemical Corp, Lakewood, NJ, USA) and 0.05 mg/mL DNAse I (Sigma-Aldrich, St. Louis, MO, USA) and incubated at 37 °C for 1 h. Pelleted cells were resuspended in 1 mL of Red Blood Cell Lysing buffer (Sigma-Aldrich) and washed. Fluorochrome-tagged monoclonal antibodies were purchased from BD Bioscience (San Jose, CA, USA) or BioLegend (San Diego, CA, USA): anti-CD3 (145–2C11, 17A2), anti-CD4 (RM4–5), anti-CD8 (53–6.7), anti-CD44 (IM7), anti-CXCR3 (CXCR3–173), anti-EOMES (Dan11mag), anti-CD11b (M1/70), anti-Ly6C (HK1.4), anti-CCR2 (475301), anti-Gr-1 (RB6.8C5), anti-Siglec F (E50–2440), and anti-CD11c (HL3). Influenza virus-specific CD8 T cells were detected using biotin-labeled H-2D^b^/NP_366–374_ (ASNENMETM) and PA_224–233_ (SSLENFRAYV) pentamers [38]. Both pentamers were purchased from ProImmune (Oxford, UK). Cells were suspended with antibodies in fluorescence-activated cell sorting (FACS) buffer (1 × PBS with 0.1% (w/v) bovine serum albumin and 0.1% (w/v) sodium azide) for 30 min at 4 °C. Intracellular staining using a mixture of the fixation and permeabilization buffers of the Foxp3 staining kit (eBioscience, San Diego, CA, USA) was followed. After staining, samples were analyzed using BD LSRFortessa, LSRII, or LSRFortessa X-20 platforms (Becton–Dickinson, Mountain View, CA, USA) running FlowJo software (Tree Star, Ashland, OR, USA).

### Intracellular cytokine staining

To measure cytokine production by CD8 T cells, 5 × 10^5^ cells were incubated with 1 µg/mL influenza virus NP_366–374_ peptide (Cosmogenetech, Seoul, Korea), 10 U/mL IL-2 (Peprotech, Princeton, NJ, USA), and 4 µL/6 mL monensin (BD Bioscience) for 5 h at 37 °C in a 5% (v/v) CO_2_ incubator. The cells were collected, stained with anti-IFN-γ (XMG1.2) and anti-TNF-α (MP6-XT22) antibodies and subjected to flow cytometry.

### Reverse-transcription quantitative PCR

Lungs were homogenized in 1 mL of TRIzol reagent (Thermo Fisher, Waltham, MA, USA). After RNA extraction, cDNA was synthesized using AccuPower CycleScript RT PreMix (Bioneer, Daejeon, Korea). PowerUp SYBRTM Green Master Mix (Applied Biosystems, Waltham, MA, USA) was used to perform real-time quantitative PCR. The primers were as follows: IP-10 (CXCL-10) forward 5ʹ-GAC GGT CCG CTG CAA CTG-3ʹ, reverse 5ʹ-CTT CCC TAT GGC CCT CAT TCT-3ʹ [39] and GAPDH, forward 5ʹ-TCA CCA CCA TGG AGA AGG C-3ʹ, reverse 5ʹ-GCT AAG CAG TTG GTG GTG CA-3ʹ [40].

### ELISA

Influenza virus-specific antibodies were measured via ELISA as described previously [41]. Ninety-six-well plates were coated with 1 µg/mL influenza A H1N1 (A/Puerto Rico/8/1934) hemagglutinin (HA; Sino Biological Inc., Wayne, PA, USA). Plates were blocked with 1 × casein solution buffer (Vector Laboratories Inc., Burlingame, CA, USA) for 2 h at 37 °C and washed with PBS-Tween 20 buffer. Then, twofold dilutions of serum samples were added, followed by incubation for 1 h and incubation with anti-mouse IgG HRP (Promega, Madison, WI, USA) for 30 min at 37 °C. The plates were developed with tetramethylbenzidine (Life Technologies, Carlsbad, CA, USA) for 10 min and absorbances at 450 nm were measured using an Epoch device (Biotek, Winooski, VT, USA).

### Plaque assay

The virus titration was measured by plaque assay [42]. Briefly, monolayers of Madin-Darby Canine Kidney (MDCK) cells were prepared in 6-well plate and incubated for 24 h. MDCK cells in culture were washed thoroughly with PBS at least twice, followed by the additional of 100 μl of lung lysate solution in serial dilution for 1 h. Infected MDCK cells were washed and added < 1% SeaPlaque™ Agarose (Lonza, Basel, Switzerland) containing 1 μg/ml of L-(tosylamido-2-phenyl) ethyl chloromethyl ketone (TPCK) -trypsin (Thermofisher, Waltham, MA, USA). After 3–4 days of incubation at 37℃, agarose was removed and plates were fixed with 4% formaldehyde solution and stained 0.5% crystal violet (Sigma-Aldrich, St. Louis, MO, USA).

### Statistical analysis

All data were analyzed as described in the figure legends using GraphPad Prism software (GraphPad, CA, USA). A *p* value < 0.05 was considered statistically significant.

### Supplementary Information

**Additional file 1**. **Supplementary figure S1.** Cells were isolated from peribronchial lymph nodes (LNs) and spleens on day 5 after influenza infection. CD8 T cells from each organ were stained with H-2Db/NP_366–374_ and H-2Db/PA_224–233_ pentamers (H-2Db/NP&PA) and analyzed via flow cytometry. Representative dot plots of virus-specific CD8 T cells are shown. The numbers in the dot plots are pentamer+ cell percentages.

## Data Availability

The datasets used and/or analyzed during the current study are available from the corresponding author on reasonable request.

## Abbreviations

I-TAC: Interferon-induced T cell α chemoattractant
IFN-γ: Interferon-γ
IL-15: Interleukin-15
IL-4: Interleukin-4
IL-4C: Interleukin-4 and an anti-interleukin-4 antibody complex
LCMV: Leukocyte choriomeningitis virus
MAIT: Mucosal-associated invariant T
MIG: Monokine induced by γ-interferon
NKT: Natural killer T
PBS: Phosphate buffered saline
PR8: Influenza A/Puerto Rico/8/34
TNF-α: Tumor-necrosis factor-α
