# Supplementary material for: IL-4/IL-4 Ab complex enhances the accumulation of both antigen-specific and bystander CD8 T cells in mouse lungs infected with influenza A virus
Source: Lab Anim Res. 2023 Dec 1;39:32. doi: 10.1186/s42826-023-00183-2 (PMC10691054; doi:10.1186/s42826-023-00183-2)
Supplement: Supplementary file 1 — Additional file 1. Supplementary figure S1. Cells were isolated from peribronchial lymph nodes (LNs) and spleens on day 5 after influenza infection. CD8 T cells from each organ were stained with H-2Db/NP366–374 and H-2Db/PA224–233 pentamers (H-2Db/NP&PA) and analyzed via flow cytometry. Representative dot plots of virus-specific CD8 T cells are shown. The numbers in the dot plots are pentamer+ cell percentages. [file 42826_2023_183_MOESM1_ESM.pdf]

IL-4/IL-4 Ab complex enhances the accumulation of both antigen-specific and bystander CD8 T cells in mouse lungs infected with influenza A virus

Hi Jung Park[1], Eun Ah Choi[1], Sung Min Choi[1], Young-Ki Choi[4], Jae Il Lee[1,2,3,*], and Kyeong Cheon Jung[1,2,5,6,*]

Supplementary Figure

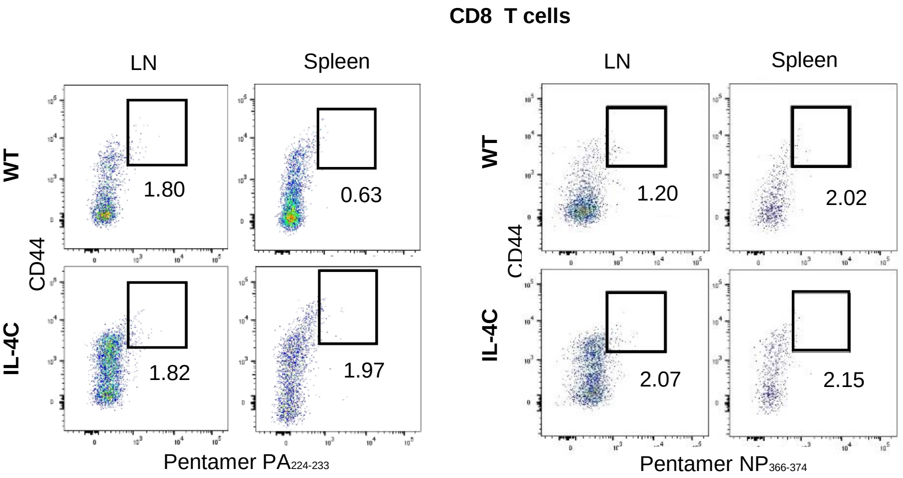

Supplementary Figure S1. Cells were isolated from peribronchial lymph nodes (LNs) and spleens on day 5 after influenza infection. CD8 T cells from each organ were stained with H-2Db/NP$_{366–374}$ and H-2Db/PA$_{224–233}$ pentamers (H-2Db/NP&PA) and analyzed via flow cytometry. Representative dot plots of virus-specific CD8 T cells are shown. The numbers in the dot plots are pentamer+ cell percentages.
